# Tumor Necrosis Factor Receptor-Associated Factor Regulation of Nuclear Factor κB and Mitogen-Activated Protein Kinase Pathways

**DOI:** 10.3389/fimmu.2018.01849

**Published:** 2018-08-09

**Authors:** Jian-Hong Shi, Shao-Cong Sun

**Affiliations:** ^1^Central Laboratory, Affiliated Hospital of Hebei University, Baoding, China; ^2^Department of Immunology, The University of Texas MD Anderson Cancer Center, Houston, TX, United States

**Keywords:** tumor necrosis factor receptor-associated factor, nuclear factor κB, mitogen-activated protein kinases, toll-like receptors, tumor necrosis factor receptors, inflammation

## Abstract

Tumor necrosis factor receptor (TNFR)-associated factors (TRAFs) are a family of structurally related proteins that transduces signals from members of TNFR superfamily and various other immune receptors. Major downstream signaling events mediated by the TRAF molecules include activation of the transcription factor nuclear factor κB (NF-κB) and the mitogen-activated protein kinases (MAPKs). In addition, some TRAF family members, particularly TRAF2 and TRAF3, serve as negative regulators of specific signaling pathways, such as the noncanonical NF-κB and proinflammatory toll-like receptor pathways. Thus, TRAFs possess important and complex signaling functions in the immune system and play an important role in regulating immune and inflammatory responses. This review will focus on the role of TRAF proteins in the regulation of NF-κB and MAPK signaling pathways.

## Introduction

Tumor necrosis factor receptor (TNFR)-associated factors (TRAFs) form a family of intracellular signaling molecules, which in mammalian cells includes six typical members (TRAF1–6) and an atypical member (TRAF7) (1, 2). The typical TRAF members share a similar secondary structure, including a homologous C-terminal domain termed TRAF domain and various numbers of zinc fingers. In addition, all TRAF members, except TRAF1, contain a RING domain located in the N-terminal region. The TRAF domain mediates oligomerization of TRAF proteins as well as their association with upstream receptors or adaptors and downstream effector proteins (1). The RING domain is best known for its function to mediate protein ubiquitination in a large family of E3 ubiquitinase ligases (3). TRAF6 is a well-characterized E3 ligase that specifically conjugates lysine (K) 63-linked polyubiquitin chains (4). Several other TRAF proteins, TRAF2, TRAF3, and TRAF5, have also been shown to possess K63-specific E3 functions, although the physiological function of their E3 activity is less well defined (1, 2, 5).

Originally identified as signaling adaptors of TNFR2 (6), the TRAF molecules are now known to mediate signal transduction from a large variety of immune receptors, including TNFR superfamily members and other cytokine receptors, pattern-recognition receptors (PRRs), and antigen receptors (1, 2). Among the major downstream pathways regulated by TRAFs are those leading to activation of the nuclear factor κB (NF-κB) and mitogen-activated protein kinases (MAPKs), which are in turn important for induction of genes associated with innate immunity, inflammation, and cell survival (7, 8). In addition to these classical functions, novel functions of TRAFs have also been discovered in recent studies. In particular, some TRAF proteins, including TRAF2 and TRAF3, function as negative regulators in some signaling pathways involved in the survival of B cells and inflammatory responses of innate immune cells (9). This review will focus on the role of TRAFs in the regulation of NF-κB and MAPK signaling pathways.

## NF-κB and MAPK Pathways

### NF-κB Pathways

Nuclear factor κB is a family of inducible transcription factors that regulate multiple biological processes, including immune and inflammatory responses, cell growth and survival, and oncogenesis (8, 10, 11). Mammalian NF-κB family includes five structurally related members, RelA, RelB, c-Rel, NF-κB1 p50, and NF-κB2 p52, which bind to a conserved DNA element (κB) as various homo- and hetero-dimers in the promoter or enhancer regions of target genes (10). NF-κBs are normally sequestered in the cytoplasm as inactive complexes bound by a family of inhibitory proteins, inhibitory κBs (IκBs), which includes IκBα, IκBβ, and several other structurally related proteins characterized by the presence of an ankyrin-repeat domain mediating binding and inhibition of NF-κBs (10). NF-κB1 and NF-κB2 are produced as precursor proteins, p105 and p100, both of which contain an IκB-like C-terminal portion and function as IκB-like molecules (12, 13). These precursor proteins can be processed by the proteasome, which involves selective degradation of the IκB-like C-terminal portion, thereby producing mature NF-κB p50 and p52, respectively, and disrupting their IκB-like function. During its translation, about half amount of p105 is constitutively processed for p50 production, whereas the remaining p105 functions as an IκB to regulate nuclear translocation of different NF-κB members, including p50, RelA, and c-Rel (13–16). In contrast to the processing of p105, the processing of p100 is tightly controlled and occurs in a signal-inducible manner (17).

Two major signaling pathways, the canonical and noncanonical pathways, mediate activation of the NF-κB members (18). The canonical pathway is based on signal-induced degradation of IκBs, particularly IκBα, which triggers nuclear translocation of p50-containing NF-κB complexes, particularly p50/RelA and p50/c-Rel heterodimers. This pathway relies on activation of a trimeric IκB kinase (IKK) complex, composed of two catalytic subunits, IKKα and IKKβ, and a regulatory subunit, NF-κB essential modulator (NEMO) (also called IKKγ). Activation of IKK is typically mediated by transforming growth factor beta-activated kinase 1 (TAK1), a MAPK kinase kinase (MAP3K) that responds to various immune receptor signals and relies on ubiquitination for its catalytic activation and signaling function (19, 20). TAK1 deficiency severely attenuates, although does not completely block, IL-1- and TNFα-induced NF-κB activation (21). Another MAP3K, MEKK3, is also involved in NF-κB activation by different inducers, such as TNFα and IL-1 (22–27). A characteristic of the canonical NF-κB signaling pathway is its rapid and transient kinetics, which is important for preventing deregulated NF-κB functions. The canonical NF-κB pathway plays an important role in regulating diverse immune functions, including innate immunity and inflammation, lymphocyte activation and differentiation, as well as immune tolerance (8, 28).

The noncanonical NF-κB pathway is based on the processing of the NF-κB2 precursor protein p100, which is triggered through site-specific phosphorylation in its C-terminal serine residues (17). This pathway is independent of TAK1 and the trimeric IKK complex but requires NF-κB-inducing kinase (NIK) and its downstream kinase IKKα (17, 18, 29). Unlike the canonical NF-κB pathway, which responds to signals from a large variety of immune receptors, the noncanonical NF-κB pathway selectively responds to a subset of TNFR superfamily members, including CD40, BAFF receptor, lymphotoxin β receptor (LTβR), RANK, TNFR2, TWEAK, CD27, etc. (30, 31). A hallmark of the noncanonical NF-κB signaling is its slow kinetics and dependence on protein synthesis (31). This is largely due to the involvement of an unusual mechanism of NIK regulation. Under normal conditions, NIK steady level is extremely low due to its constitutive degradation *via* the ubiquitin/proteasome pathway, which prevents induction of p100 processing (32). Signal-stimulated noncanonical NF-κB signaling involves stabilization of the *de novo* synthesized NIK, thereby allowing the accumulated NIK to activate IKKα and induce p100 processing. The noncanonical NF-κB pathway is best known for its role in regulating lymphoid organ development, B cell maturation, and osteoclast differentiation. However, recent studies have uncovered additional functions of this pathway and linked this pathway with autoimmune and inflammatory diseases (18).

### MAPK Pathways

Mitogen-activated protein kinases form a large family of serine/threonine kinases that respond to diverse extracellular and intracellular stimuli and mediate multiple biological processes, such as cell growth and differentiation, immune and inflammatory responses, and oncogenesis (7, 33). The mammalian MAPK family includes three subfamilies: the extracellular signal-regulated kinases (ERKs), the c-Jun N-terminal kinases (JNKs), and the p38 MAPKs (7, 33). Signal transduction of the MAPKs share a common mechanism, in which an MAPK is phosphorylated and activated by an MAPK kinase (MKK), and the MKK is in turn phosphorylated and activated by an MAP3K. Different MAPKs are regulated by distinct MKKs: MKK1 and MKK2 (also called MEK1 and MEK2) for ERK1 and ERK2, MKK4 and MKK7 for JNKs, and MKK3 and MKK6 for p38. The MAP3Ks for the MAPK signaling cascades vary among different stimulating receptors. In the immune system, MAPK signaling cascades have been extensively studied in innate immune cells stimulated *via* the PRRs, particularly the toll-like receptors (TLRs), where MAPKs mediate induction of various proinflammatory cytokines and chemokines required for host defense and inflammation (7).

In the TLR pathway, TAK1 is a primary MAP3K mediating activation of the JNK and p38 signaling cascades, whereas Tpl2 is the MAP3K mediating activation of the ERK1/2 cascade (34). The TLR-stimulated MAPK signaling involves an interesting crosstalk with the IKK/NF-κB pathway. The NF-κB1 precursor protein p105 forms a stable complex with the MAP3K Tpl2, in which p105 both stabilizes Tpl2 and prevents its phosphorylation of downstream kinases, MKK1/2 (35–39). Upon activation by the TLR signal, IKK phosphorylates p105 to induce its ubiquitin-dependent degradation, which allows the liberated Tpl2 to phosphorylate MKK1/2, leading to activation of ERK1/2. This signaling event is transient, since activated Tpl2 is rapidly degraded due to its instability when dissociated from p105. Therefore, in the TLR pathway, TAK1 functions as a master kinase mediating activation of not only the JNK and p38 MAPK cascades but also the IKK–Tpl2–ERK signaling axis.

## TRAF6 as a Mediator of NF-κB and MAPK Activation

### IKK/MAPK Activation

TRAF6 is a prototype of RING domain-containing E3 ubiquitin ligase that functions together with a dimeric E2, composed of Ubc13 and Uev1A, specifically catalyzing the synthesis of K63-linked polyubiquitin chains (4, 19). Accumulating studies have demonstrated a crucial role for TRAF6 in mediating signaling from various TNFRs as well as other immune receptors, such as the IL-1 receptor (IL-1R), IL-17R, TLRs, RIG-I-like receptors, TGFβR, and antigen receptors (20, 40–44). The C-terminal TRAF domain of TRAF6 interacts with a conserved sequence motif, Pro-X-Glu-X-X-(aromatic/acidic residue), present in specific members of the TNFR superfamily, including CD40 and receptor activator of nuclear factor kappa-B (45). Through this molecular interaction, TRAF6 is recruited to the TNFRs in response to ligand stimulation, which is essential for triggering TRAF6 activation and signal transduction. The TRAF6-binding motifs are also present in signaling adaptors of other immune receptors, such as the IL-1R-associated kinases (IRAKs) of the IL-1R and TLR pathways, the mitochondrial antiviral signaling protein of the RIG-I pathway, and Act1 of the IL-17R pathway (41, 44, 46).

**Figure 1:**
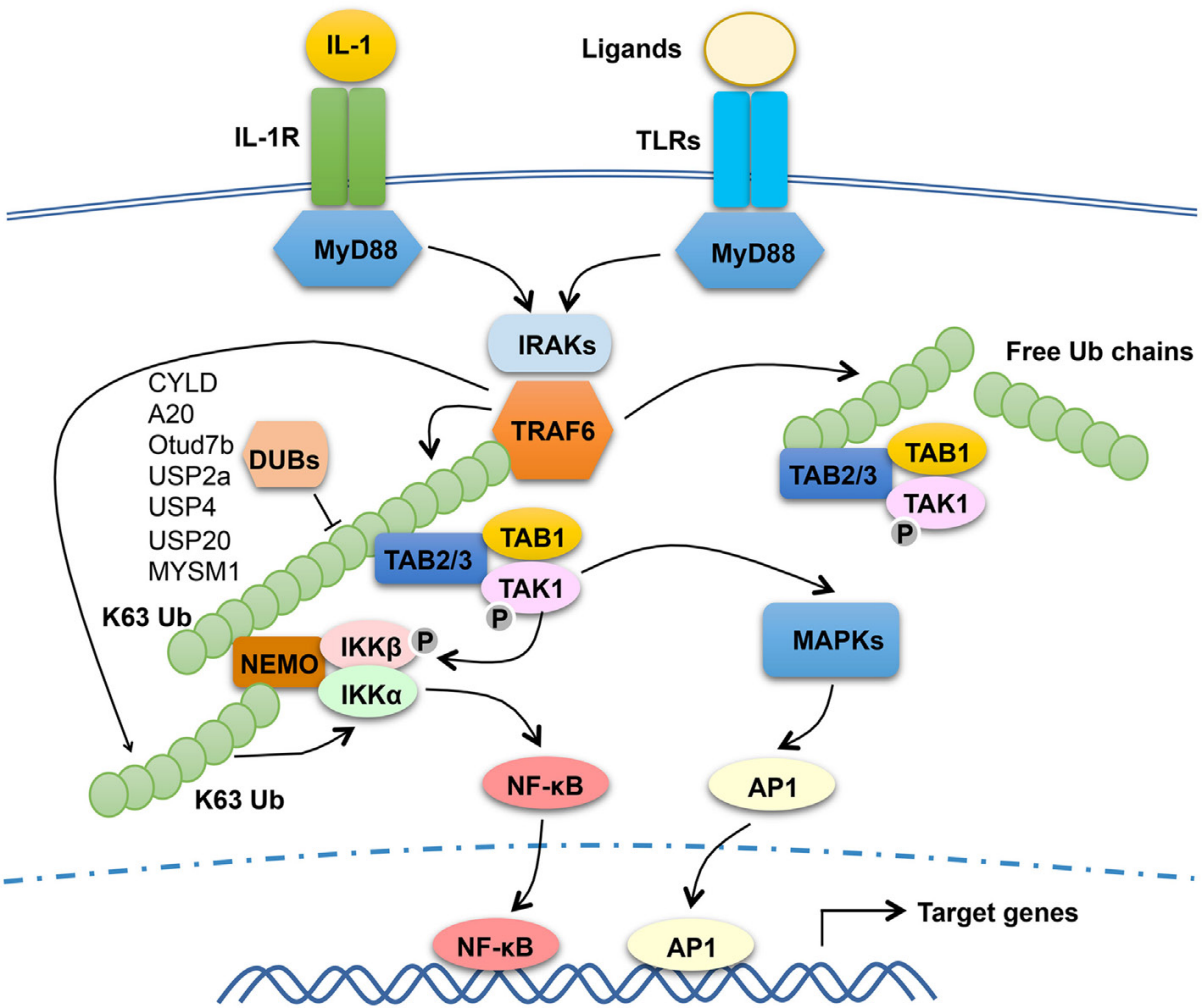
The function and regulation of tumor necrosis factor receptor-associated factor (TRAF)6 in MyD88 signaling pathway. Upon stimulation with IL-1 or toll-like receptor (TLR) ligands, MyD88 recruits IL-1R-associated kinases (IRAKs) (including IRAK1, IRAK2, and IRAK4) and TRAF6 to assemble a MyD88 signaling complex. Once activated in the MyD88 complex, TRAF6 functions as an E3 ubiquitin ligase that catalyzes the synthesis of K63-linked polyubiquitin chains conjugated to itself or NF-κB essential modulator (NEMO) or existing as free ubiquitin chains. The self-ubiquitinated TRAF6 recruits the ubiquitin-dependent kinase transforming growth factor beta-activated kinase 1 (TAK1) and its downstream kinase IκB kinase (IKK) to assemble a signaling complex that facilitates TAK1 and IKK activation. This process requires the TAK1 regulatory subunit TAB 2 (or TAB 3) and the IKK regulatory subunit NEMO, both have ubiquitin-binding functions. Activated TAK1 mediates activation of IKK and mitogen-activated protein kinases (MAPKs), which further activate nuclear factor κB (NF-κB) (RelA- and c-Rel-containing complexes) and AP1. TRAF6 mediated NEMO ubiquitination also contributes to the activation of IKK and NF-κB. Several DUBs have been shown to negatively regulate TRAF6 function through deconjugation of its K63 polyubiquitin chains.

The signaling mechanism of TRAF6 has been extensively studied in the TLR and IL-1R pathways (Figure 1). TLRs transduce signals *via* common adaptors, MyD88 and TRIF (47). With the exception of TLR3, which signal *via* TRIF, all TLRs, as well as IL-1R, rely on MyD88 for signal transduction, although TLR4 signals through both MyD88 and TRIF. Upon ligand binding, MyD88-dependent TLRs recruit IRAKs (including IRAK1, IRAK2, and IRAK4) *via* the adaptor MyD88 to trigger the formation of a receptor-associated signaling complex that also contains TRAF6 (Figure 1). Once activated in the MyD88 signaling complex, TRAF6 functions as an E3 ubiquitin ligase that conjugates K63-linked ubiquitin chains onto itself as well as to other proteins, such as IRAK1 and the IKK regulatory subunit, NEMO (19, 20). Precisely how TRAF6 mediates activation of downstream pathways is incompletely understood. It is generally thought that self-ubiquitinated TRAF6 recruits the downstream kinases TAK1 and IKK to assemble a signaling complex that facilitates TAK1 and IKK activation. In support of this model, both TAK1 and IKK contain regulatory subunits that bind K63-linked polyubiquitin chains (48–50). The TAK1 complex contains two regulatory subunits, TAB 1 and TAB 2 (or TAB 3), and TAB 2 and TAB 3 both contain an Npl4 Zinc Finger type of ubiquitin-binding domain that specifically binds K63-linked polyubiquitin chains (48, 51). The regulatory subunit of IKK, NEMO, also contains a ubiquitin-binding domain, called CC2-LZ (coiled-coil2-lucine zipper) or UBAN (ubiquitin binding in ABIN and NEMO proteins), capable of binding K63 ubiquitin chains (49, 50, 52–54). In further support of this model, mutation of an autoubiquitination site, K124, of TRAF6 attenuates its function in mediating TAK1/IKK activation (55). However, a subsequent study suggests that the E3 ligase activity of TRAF6, although required for TAK1 activation, is dispensable for TRAF6 association with the TAK1 complex (56). Moreover, this study also suggests that TRAF6 autoubiquitination is dispensable for activation of TAK1 and its downstream NF-κB and MAPK pathways by IL-1 and RANKL, whereas TRAF6-mediated NEMO ubiquitination contributes to the activation of IKK and NF-κB (56). Since unconjugated ubiquitin chains are able to activate TAK1 (57), it is also likely that both conjugated and unconjugated K63 ubiquitin chains within the TRAF6/TAK1 complex contribute to TAK1 activation.

Although the E3 ubiquitin ligase activity of TRAF6 is generally believed to be essential for its signaling function, there are controversies. A recent study employing both cell line reconstitution and knockin mouse approaches demonstrates that inactivation of the E3 ligase activity of TRAF6 only partially inhibits its function in mediating activation of NF-κB and MAPK signaling stimulated by IL-1, TLRs, and RANKL (58). It seems that the Pellino family of E3 ligases, particularly Pellino1 and Pellino2, could compensate the E3 function of TRAF6 to mediate K63 ubiquitin chain conjugation for TAK1 activation (58). Since complete deletion of TRAF6 abolishes MyD88-dependent activation of NF-κB and MAPK signaling, these findings suggest that in addition to serving as an E3 ligase, TRAF6 may also function an adaptor.

### TRAF6 Regulation

Given the crucial role of TRAF6 in mediating activation of NF-κB and MAPK pathways and inflammation, it is not surprising that the function of TRAF6 is subject to tight control by various regulators (Figure 1). An early study suggests that IL-1-stimulated TRAF6 polyubiquitination, an indicator of TRAF6 activation, occurs transiently suggesting its regulation by deubiquitination (59). Several DUBs have been implicated in the regulation of TRAF6 activation through deconjugation of its K63 polyubiquitin chains; these include CYLD, A20, Otud7b (also called Cezanne), USP2a, USP4, USP20, and myb-like SWIRM and MPN domain 1 (60–66). Another DUB, YOD1 (also called Otud2), inhibits TRAF6 ubiquitination and signaling function *via* a non-catalytic mechanism that involves binding to the C-terminal TRAF domain of TRAF6 and, thereby, preventing TRAF6 interaction with an activating adaptor protein, p62 (also called Sequestosome-1) (67). It is unclear why there are so many DUBs involved in TRAF6 regulation, but it is likely that they function in different cell types and/or distinct receptor pathways. It is also important to note that the role of some TRAF6-regulating DUBs in regulating TLR signaling and innate immunity is yet to be demonstrated using *in vivo* approaches.

TRAF6 regulation also involves various other factors. A member of the NOD-like receptors (NLR) family, NLRC3, has been shown to inhibit signaling from MyD88-dependent TLRs (68). NLRC3 interacts with and inhibits K63 ubiquitination of TRAF6, thereby negatively regulating TLR-stimulated NF-κB activation. Interestingly, NLRC3-mediated TRAF6 regulation does not influence MAPK signaling pathways (68), indicating a different role for K63 ubiquitination of TRAF6 in regulating NF-κB and MAPK pathways. The molecular mechanism by which NLRC3 inhibits K63 ubiquitination and signaling function of TRAF6 is undefined. A putative E2 molecule, Ube2o binds to TRAF6 and inhibits TRAF6 K63 ubiquitination and activation of NF-κB in the IL-1β and LPS pathways (69). This function of Ube2o is independent of its ubiquitin-conjugating domain and appears to act through disruption of TRAF6 binding to MyD88. TRAF6 is also regulated by a protein kinase, MST4, which phosphorylates TRAF6 at two threonine residues (T463 and T486) in the C-terminal TRAF domain and inhibits the oligomerization and autoubiquitination activity of TRAF6 (70). MST4 knockdown promotes TLR signaling and cytokine induction in cell culture and sensitizes mice to septic shock induction (70). In contrast to the negative role of MST4 in TRAF6 regulation, another kinase, RSK2, positively regulates TRAF6 function and LPS- and IL-1β-stimulated activation of MAPKs and NF-κB (71). RSK2 phosphorylates TRAF6 at three N-terminal serines (S46, S47, and S48), thereby promoting the K63 ubiquitination and signaling function of TRAF6 (71). Future studies should examine whether these negative (T463 and T486) and positive (S46, S47, and S48) phosphorylation sites are phosphorylated *in vivo* along with TLR stimulation.

## TRAF2 in TNFR Signaling

**Figure 2:**
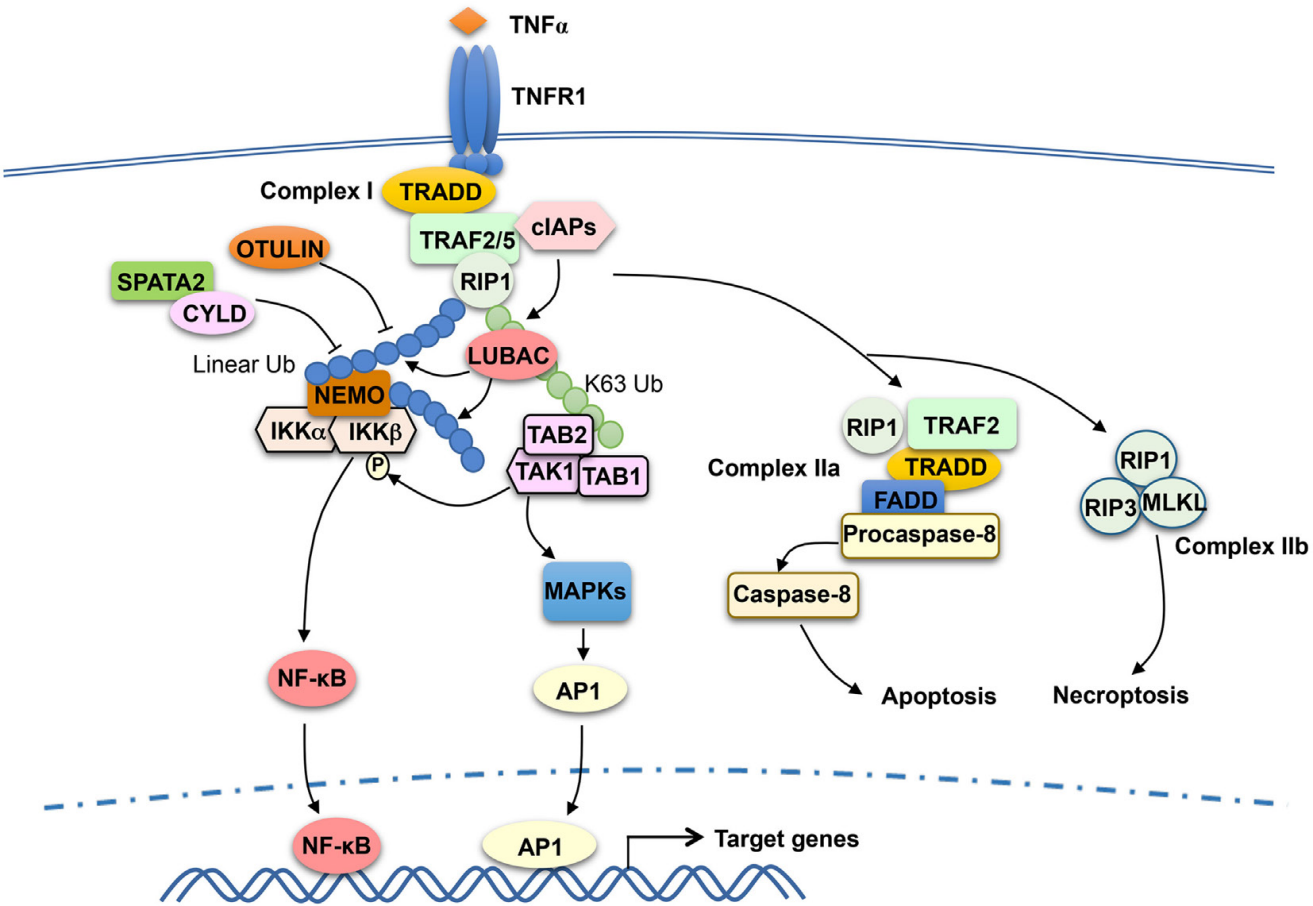
Tumor necrosis factor receptor-associated factor (TRAF)2 and TRAF5 in tumor necrosis factor receptor (TNFR)1 signaling. TNFα binding to TNFR1 triggers the assembly of a TNFR1-associated signaling complex, complex I, which is composed of TNF receptor-associated death domain (TRADD), receptor-interacting protein kinase 1 (RIP1), TRAF2 or TRAF5 (TRAF2/5), and the E3 ubiquitin ligases cIAP1 and cIAP2 (cIAPs). Upon activation, cIAPs conjugate K63-linked ubiquitin chains to RIP1, which facilitates recruitment and activation of the kinase transforming growth factor beta-activated kinase 1 (TAK1) as well as the recruitment of the linear ubiquitin ligase complex LUBAC. LUBAC conjugates linear ubiquitin chains to RIP1 and, thereby, facilitates the requirement of IκB kinase (IKK) *via* the linear ubiquitin-binding function of NF-κB essential modulator (NEMO). Subsequent ubiquitination of NEMO by LUBAC, along with TAK1-mediated IKKβ phosphorylation, results in IKK activation. The activated IKK and TAK1 mediate activation of nuclear factor κB (NF-κB) and mitogen-activated protein kinase (MAPK)/AP1 signaling pathways that promote cell survival. TRAF2 also participates in the subsequent formation of a cytoplasmic complex, complex IIa, which mediates apoptosis induction. When caspase-8 is inhibited, TNFR1 signaling also leads to formation of complex IIb, leading to necroptosis. DUBs, including OTULIN and CYLD, negatively regulate signaling functions of LUBAC by cleaving linear ubiquitin chains. SPATA2, as a high-affinity binding partner of CYLD and LUBAC, facilitates CYLD function by recruiting CYLD to LUBAC.

TRAF2 has been extensively studied as a mediator of TNFR1 signaling and known to be crucial for TNFα-stimulated NF-κB and MAPK pathways. TRAF2 is essential for TNFα-induced activation JNK, although it is functionally redundant with TRAF5 in the activation of NF-κB (72, 73). TRAF2 does not directly bind TNFR1, but it can be recruited to TNFR1 *via* the adaptor protein TNF receptor-associated death domain (TRADD) (74, 75). The cytoplasmic tail of TNFR1 contains a death domain that mediates interaction with the death domain of TRADD, and TRADD binds to TRAF2 *via* the N-terminal region of TRADD and the C-terminal TRAF domain of TRAF2 (74, 76). TNFR1 signaling involves sequential formation of two different complexes, complex I and complex II, which mediate cell survival and cell death, respectively (77) (Figure 2). Complex I is associated with TNFR1 and composed of TRADD, TRAF2 and its close homolog TRAF5, receptor-interacting protein kinase 1 (RIP1), and the E3 ubiquitin ligases cIAP1 and cIAP2 (77). In response to TNFR1 stimulation, cIAP1 and cIAP2 conjugates K63-linked ubiquitin chains to RIP1, which facilitates the recruitment and activation of TAK1 and IKK kinase complexes leading to activation of NF-κB and induction of survival genes (49, 78–80). Following the initial signaling, complex I dissociates from TNFR1 leading to formation of the cytoplasmic complex IIa, composed of TRAF2, RIP1, TRADD, FADD, and procaspase-8. This second complex triggers activation of Caspase-8 and apoptosis, which serves as a checkpoint mechanism mediating cell death when complex I-mediated NF-κB activation fails (77). TNFR1 also induces a complex (complex IIb), composed of RIP1, RIP3, and MLKL, which mediates cell death *via* necroptosis (81).

Like TRAF6, TRAF2 contains an N-terminal RING domain and has been implicated as a K63-specific E3 ubiquitin ligase (82). However, the RING domain structure of TRAF2 differs significantly from that of TRAF6, and TRAF2 does not interact with Ubc13 and several other E2s (83). A subsequent study suggests that the E3 ligase function of TRAF2 requires a cofactor, sphingosine-1-phosphate (S1P), which binds to the RING domain of TRAF2 and stimulates TRAF2-mediated K63 ubiquitination of RIP1 (84). TRAF2 also interacts with sphingosine kinase 1 (SphK1), one of the isoenzymes catalyzing the generation of S1P, and siRNA-mediated SphK1 silencing attenuates TNFα-stimulated RIP1 ubiquitination and NF-κB activation (84, 85). Whether TRAF2 functions as an adaptor or an E3 ligase in the TNFR1 pathway is still in debate. While TRAF2 has been implicated as an E3 that mediates TNFα-induced RIP1 ubiquitination and NF-κB activation (84, 86), other studies suggest that the RING domain of TRAF2 is dispensable for its function in mediating TNFα-induced canonical NF-κB signaling (87). It is generally believed that the TRAF2-recruited cIAPs function as E3 ligases of RIP1 in the TNFR1 complex I (Figure 2). The cIAP-binding motif, but not RING domain, of TRAF2 is essential for TNFR1-stimulated RIP1 ubiquitination and canonical NF-κB activation (87). cIAP1 and cIAP2 directly ubiquitinate RIP1 *in vitro* and are required for TNFα-induced RIP1 ubiquitination and NF-κB activation *in vivo* (78–80).

TRAF2 is functionally redundant with TRAF5 in mediating TNFα-stimulated NF-κB activation. Mouse embryonic fibroblasts (MEFs) deficient in either TRAF2 or TRAF5 are fully functional in TNFα-stimulated NF-κB activation, whereas MEFs lacking both TRAF members are severely attenuated, although not completely defective, in the NF-κB activation (73). These findings confirm the important role of TRAF2 and TRAF5 in TNFα-stimulated NF-κB signaling and also suggest the involvement of additional mechanisms. The role of cIAPs is cell-type specific. shRNA-mediated knockdown of cIAP1 in skeletal myoblasts largely blocked TNFα-stimulated NF-κB signaling, whereas simultaneous knockdown or knockout of cIAP1 and cIAP2 is required for blocking NF-κB activation in MEFs (79, 88).

Recent studies suggest that the signaling function of TNFR1 complex I also involves another E3 ubiquitin ligase, LUBAC, which specifically conjugates linear ubiquitin chains (89, 90). LUBAC is a complex composed of three subunits: SHARPIN, HOIL-1L, and HOIP. In response to TNFR1 stimulation, LUBAC is recruited to the TNFR1 complex I *via* binding to K63 ubiquitin chains conjugated to RIP1 by the cIAPs (89) (Figure 2). Within the TNFR1 complex, LUBAC mediates linear ubiquitination of several components of the TNFR1 complex 1, including RIP1 and the IKK regulatory subunit NEMO (91, 92). The LUBAC-catalyzed synthesis of linear ubiquitin chains plays an important role in recruitment and activation of IKK. Unlike TAB 2 and TAB 3, which specifically binds to K63 ubiquitin chains, NEMO preferentially binds to linear ubiquitin chains *via* its ubiquitin-binding domain, UBAN (53, 93–95). The K63 ubiquitin- and linear ubiquitin-conjugated RIP1 molecules recruit TAK1 and IKK complexes, respectively, thereby facilitating IKK activation by TAK1 (Figure 2). Linear ubiquitination of NEMO may directly contribute to IKK activation. It has been proposed that linear ubiquitin chain-conjugated NEMO can be bound by another NEMO molecule, which promotes IKK dimerization and catalytic activation (96). It has also been suggested that NEMO binding to linear ubiquitin chains may cause a conformational change in IKK, thereby facilitating IKKβ phosphorylation by TAK1 (97). The signaling function of LUBAC is negatively regulated by DUBs capable of cleaving linear ubiquitin chains, including OTULIN and CYLD (98–100). In addition, the DUB A20 is recruited to the TNFR1 complex *via* binding to linear ubiquitin chains and contributes to the inhibition of NF-κB activation possibly by preventing NEMO recruitment (101, 102).

TRAF2 is also a mediator of TNFR2 signaling. In fact, TRAF2, along with TRAF1, was originally identified as signaling adaptors physically associated with TNFR2 (6). TRAF2 binds to the cytoplasmic domain of TNFR2 and recruits E3 ubiquitin ligases cIAP1 and cIAP2 to the TNFR2 signaling complex, which is important for TNFR2-stimulated NF-κB activation (103, 104). The cytoplasmic tail of TNFR2 contains sequence motifs that are directly bound by TRAF2 (6, 105, 106). TRAF2 also mediates activation of NF-κB and MAPK pathways triggered by other TNFR superfamily members, such as CD40, OX40, 4-1BB, LTβR, and GITR (107–111).

## TRAF Control of Noncanonical NF-κB Pathway

While TRAF proteins are generally known as signaling adaptors that mediate activation of NF-κB and MAPK pathways, it is now clear that TRAF2 and TRAF3 are pivotal negative regulators of the noncanonical NF-κB pathway (18). An initial study identified TRAF3 as a protein physically interacting with the noncanonical NIK and mediating ubiquitin-dependent NIK degradation (32). TRAF3 knockdown causes NIK stabilization and induction of p100 processing, and signal-induced noncanonical NF-κB activation is associated with TRAF3 degradation and concomitant accumulation of NIK, suggesting that the TRAF3-mediated NIK degradation is a central mechanism of noncanonical NF-κB regulation (32). This finding is corroborated by subsequent studies using TRAF3 knockout mice demonstrating that TRAF3 deficiency causes NIK accumulation and noncanonical NF-κB activation (112–114). Domain mapping analyses revealed an N-terminal motif of NIK, ISIIAQA (amino acids 78–84), which is required for NIK–TRAF3 interaction. A NIK mutant harboring deletion of this motif, named NIKΔ78–84 or NIKΔT3, is stable due to impaired interaction with TRAF3 (32). Transgenic expression of this NIK mutant in B cells causes maximal noncanonical NF-κB activation and B cell survival independently of BAFF, resulting in drastic B cell hyperplasia (115).

Unlike TRAF3, TRAF2 does not bind the N-terminal region of NIK and only weakly interacts with NIK *via* a C-terminal region (32, 116). Interestingly, however, TRAF2 deficiency also causes noncanonical NF-κB activation (114, 117). Moreover, the E3 ubiquitin ligases cIAP1 and cIAP2 were later on found to mediate NIK ubiquitination and degradation (118, 119). Biochemical and genetic evidence suggests that TRAF2, TRAF3, and the cIAPs function together as an E3 ubiquitin ligase complex mediating NIK ubiquitination (120, 121). It has been proposed that within this complex, TRAF2 and TRAF3 bind cIAPs and NIK, respectively, and recruit NIK to the E3 ligases cIAPs *via* TRAF2–TRAF3 dimerization (31, 120, 121). These findings provide mechanistic insight into the functions of small molecule IAP antagonists in cancer treatment. Recent studies suggest that the IAP antagonists not only induce cancer cell death but also promote antitumor immunity and synergize with immune checkpoint inhibitors in mouse models of cancer immunotherapy (122–125). The immunostimulatory action of IAP antagonists is likely due to activation of the NIK-dependent noncanonical NF-κB activation. In addition, the IAP antagonists may also activate innate immune cells in tumor microenvironment. As will be discussed in the following section, disruption of the TRAF–cIAP E3 complex in macrophages promotes production of M1 type of proinflammatory cytokines that facilitate recruitment of antitumor effector T cells to the tumor microenvironment (126).

Precisely how the TRAF–cIAP complex is assembled and regulates NIK stability is incompletely understood. More recent studies suggest that another TRAF member, TRAF1, may also be involved in cIAP E3 complex function and NIK regulation (110, 127). TRAF1 and TRAF2 forms a heterotrimer, TRAF1:(TRAF2)2, which binds cIAP2 more strongly than TRAF2 (127). Since TRAF1 is induced by various cellular stimuli, it may function as a modifier of the TRAF–cIAP complex under certain conditions. In support of this idea, TRAF1 appears to play a role in restraining TCR-stimulated noncanonical NF-κB activation (110). TRAF1 deletion allows murine CD8 T cells to respond to TCR stimulation, in the absence of 4-1BB costimulation, for the activation of noncanonical NF-κB (110). However, the role of TRAF1 in NF-κB signaling is controversial, since another study suggests that TRAF1 directly binds NIK and stabilizes NIK by interfering with NIK association with the TRAF2–cIAP2 complex under overexpression conditions (128). It is important to note, though, the TRAF1–NIK binding is substantially weaker than the TRAF3–NIK interaction (32), and additional studies are required to examine the physiological role of TRAF1 in noncanonical NF-κB regulation.

## Anti-Inflammatory Function of TRAF2 and TRAF3

### Anti-Inflammatory Function

Compared to TRAF6, much less is known about the function of TRAF2 and TRAF3 in regulating TLR signaling. Nevertheless, recent studies suggest a role for both TRAF2 and TRAF3 in negatively regulating TLR-stimulated expression of proinflammatory cytokines (9). TRAF3 deficiency promotes proinflammatory cytokine induction, while inhibits type I interferon induction, in macrophages (129, 130). The anti-inflammatory function of TRAF3 has been demonstrated using myeloid cell-conditional TRAF3 knockout (TRAF3-MKO) mice (126, 131). Myeloid cell-specific deletion of TRAF3 does not affect macrophage differentiation, but renders mice hypersensitive to colitis induction in the dextran sodium sulfate (DSS) model (126). The TRAF3-MKO mice also produce elevated levels of IgG3 and IgG2b antibody isotypes in response to T-independent and T-dependent antigens, respectively, likely due to aberrant production of proinflammatory cytokines, such as IL-12 and IL-6 (131). Consistently, the TRAF3 deficiency sensitizes macrophages to *in vitro* induction of proinflammatory cytokines by the TLR4 and TLR3 ligands LPS and polyIC (126, 131). At older ages (15–22 months), the TRAF3-MKO mice spontaneously develop chronic inflammation in multiple organs (131). Interestingly, some of these mice also develop tumors originating from both myeloid cells (histiocytes) and other cell types (including B cells and hepatocytes) that are competent in TRAF3 expression (131). These findings suggest that chronic inflammation in these mutant mice may contribute to the tumorigenesis, although it is also possible that TRAF3 may regulate a tumor-suppressive function of myeloid cells.

An unexpected finding is the anti-inflammatory function of TRAF2 in the TLR signaling pathway (126). Like TRAF3, TRAF2 negatively regulate induction of proinflammatory cytokines by the TLR ligands LPS and polyIC as well as by the cytokine IL-1β, and the myeloid cell-conditional TRAF2 knockout (TRAF2-MKO) mice are hypersensitive to colitis induction in the DSS model (126). The protective role of TRAF2 in colon inflammation has also been revealed using TRAF2 germline knockout mice, which spontaneously develop colitis (132). In addition to its anti-inflammatory role in myeloid cells, TRAF2 appears to inhibit inflammation through protecting intestinal epithelial cells from TNF-induced apoptosis (9, 132). A potential role for TRAF2 and TRAF3 in regulating human inflammatory bowel disease (IBD) is indicated by the finding that the expression level of these TRAF members is upregulated in the colonic tissue of IBD patients (133–135). However, whether the upregulation of TRAF2 and TRAF3 serves as a feedback mechanism to suppress inflammation in the human patients is unclear.

### Signaling Mechanism

**Figure 3:**
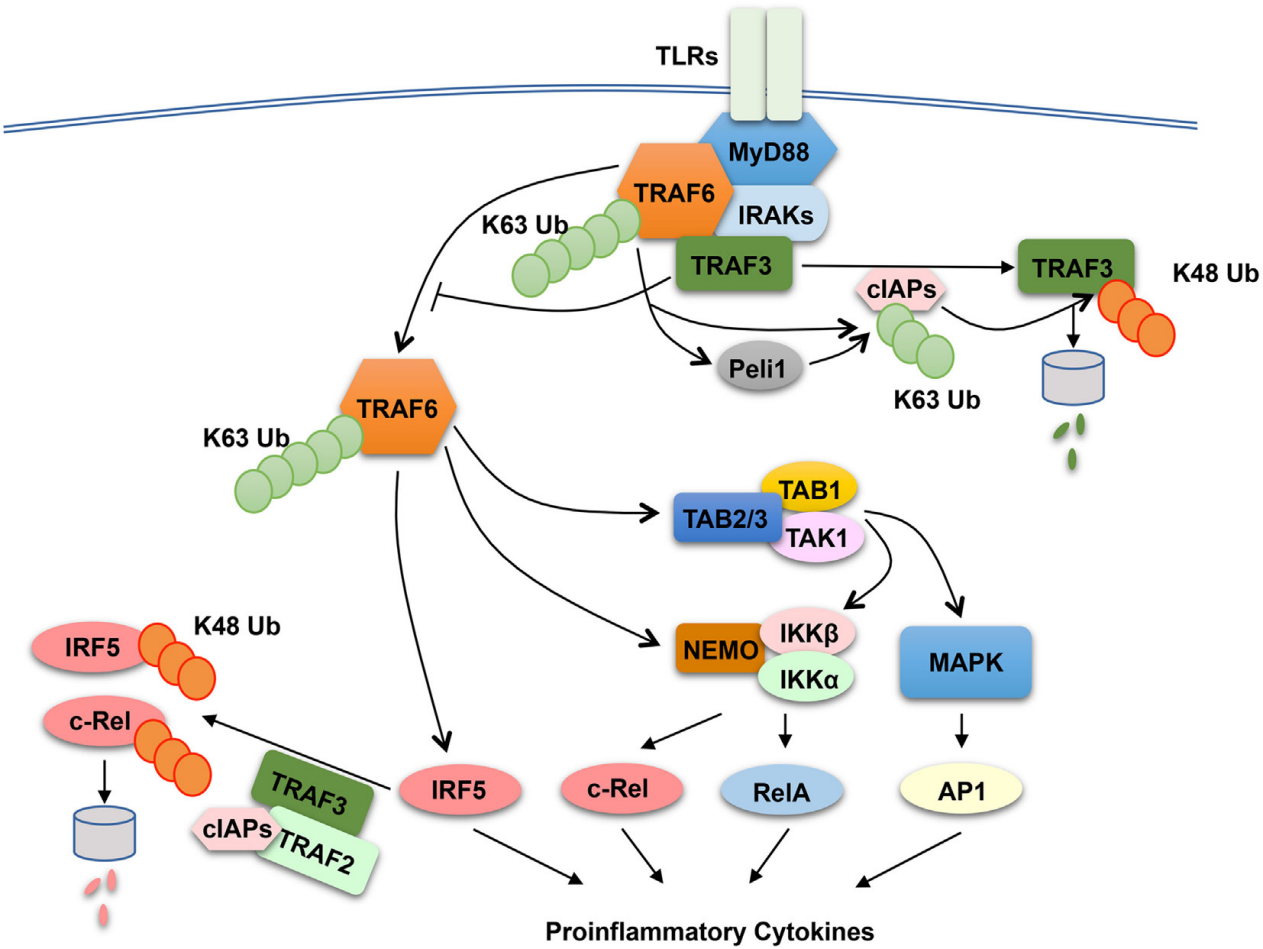
Anti-inflammatory function of tumor necrosis factor receptor-associated factor (TRAF)2 and TRAF3 in toll-like receptor (TLR) signaling pathway. In responds to TLR stimulation, TRAF6 is recruited to the MyD88 signaling complex. Upon activation, the TRAF6 complex relocates from the plasma membrane to the cytoplasm, a signaling step required for activation of the downstream nuclear factor κB and mitogen-activated protein kinase (MAPK) pathways. TRAF3 negatively regulates TLR-stimulated proinflammatory cytokine induction *via* two potential mechanisms. The first is to target the MyD88–TRAF6 complex and prevents the relocation of TRAF6 to the cytoplasm, and the second is to function together TRAF2 and cIAPs to mediate ubiquitin-dependent degradation of two major proinflammatory transcription factors, c-Rel and interferon regulatory factor 5 (IRF5). TLR signaling temporarily overrides the negative signaling function of TRAF3 by inducing TRAF3 proteolysis. In this process, TRAF6 activates cIAPs (cIAP1 and cIAP2) through K63 ubiquitination, which in turn function as E3 ubiquitin ligases to mediate K48-linked ubiquitination and proteolysis of TRAF3. In microglia, Peli1 appears to cooperate with TRAF6 or function downstream of TRAF6 to mediate cIAP activation.

The finding that both TRAF2 and TRAF3 negatively regulate proinflammatory cytokine induction by TLRs raises the question of whether these TRAF members have a common mechanism of action. TRAF3 has been shown to play a role in regulating receptor-proximal signaling in the MyD88 pathway (136) (Figure 3). In response to TLR stimulation, both TRAF6 and TRAF3 are recruited to the MyD88 signaling complex, in which TRAF3 appears to prevent the relocation of the TRAF6 complex from the membrane to the cytoplasm, a signaling step required for activation of the downstream MAPK pathways (136). Interestingly, TLR signaling induces TRAF3 degradation, which is important for optimal activation of MAPKs (136). TRAF3 degradation appears to involve TRAF6-mediated K63 ubiquitination and activation of the E3 ubiquitin ligases cIAP1 and cIAP2, which in turn conjugate K48-linked ubiquitin chains to TRAF3 and cause ubiquitin-dependent TRAF3 degradation in the proteasome (136) (Figure 3). TRAF3 degradation also occurs in microglia, resident macrophages of the central nervous system (CNS) (137). In microglia, TRAF3 undergoes degradation and resynthesis along with induction of experimental autoimmune encephalomyelitis (EAE), an animal model of the neuroinflammatory disease multiple sclerosis (137). The TRAF3 degradation also requires the E3 ubiquitin ligase Peli1, which is abundantly expressed in microglia. Peli1 deficiency blocks TRAF3 degradation and causes its accumulation, which contributes to attenuated induction of proinflammatory cytokines and chemokines in microglia and ameliorated EAE pathogenesis (137). Peli1 appears to cooperate with TRAF6 or function downstream of TRAF6 to mediate cIAP activation, since Peli1 deficiency inhibits TRAF6-mediated induction of cIAP ubiquitination (137) (Figure 3). The role of TRAF3 in suppressing CNS inflammation also involves negative regulation of IL-17R signaling (138). TRAF3 binds to IL-17R and interferes with the formation of the IL-17R–Act1–TRAF6 signaling complex and IL-17-stimulated activation of NF-κB and MAPK pathways. Nuclear Dbf2-related kinase 1 inhibits the binding of TRAF3 to IL-17R and, thereby, promotes IL-17R signaling and inflammation (139).

The role of TRAF2 and TRAF3 in MAPK regulation may be cell type specific, since bone marrow-derived macrophages and peritoneal macrophages derived from TRAF2-MKO and TRAF3-MKO mice do not display hyper-activation of MAPKs upon LPS stimulation (126, 131). Another potential mechanism that underlies the anti-inflammatory function of TRAF2 and TRAF3 is suppression of noncanonical NF-κB activation. As discussed in above sections, TRAF2 and TRAF3 are both essential components of the TRAF-cIAP E3 ubiquitin ligase that mediates NIK degradation in noncanonical NF-κB pathway (31). Deficiency in either TRAF2 or TRAF3 in macrophages causes constitutive activation of noncanonical NF-κB, as shown by NIK accumulation and p100 processing (126, 131). However, the noncanonical NF-κB activation does not seem to play an important role, since deletion of NIK in the TRAF2-deficiency macrophages fails to prevent LPS-stimulated hyper-expression of proinflammatory cytokine genes, except a partial inhibition of *Il23a* induction (126). It appears that TRAF2 and TRAF3 negatively regulate two transcription factors, c-Rel and interferon regulatory factor 5 (IRF5), which belong to the NF-κB and IRF families, respectively (126). IRF5 is activated by TRAF6 in the MyD88 signaling pathway, whereas c-Rel is activated by IKK through phosphorylation-dependent degradation of the NF-κB inhibitor IκBα (10, 18). Both c-Rel and IRF5 are crucial mediators of TLR-stimulated proinflammatory cytokine expression (140–145). TRAF2 and TRAF3 control the steady level expression of these transcription factors posttranslationally, which involves a ubiquitin- and proteasome-dependent mechanism (Figure 3). In wild-type macrophages, c-Rel and IRF5 undergo constitutive degradation, and this process is requires TRAF2 and TRAF3, since deletion of either TRAF2 or TRAF3 causes stabilization and accumulation of c-Rel and IRF5 (126). The constitutive degradation of c-Rel and IRF5 also involves cIAPs, since incubation of macrophages with a small molecule IAP antagonist results in accumulation of c-Rel and IRF5. These findings suggest the possibility that the TRAF–cIAP E3 ubiquitin ligase mediates degradation of c-Rel and IRF5 in addition to NIK (Figure 3).

Emerging evidence suggests that the anti-inflammatory function of TRAF–cIAP complex in macrophages plays an important role in regulating antitumor immunity. Myeloid cell-specific deletion of TRAF2 promotes the generation of M1 type of macrophages in tumor microenvironment, which promotes recruitment of CD4 and CD8 effector T cells and enhances antitumor immunity (126). Consistently, as discussed in the above section, IAP antagonists promote antitumor immunity and synergize with immune checkpoint inhibitors in mouse models of cancer immunotherapy (122–125). These immunostimulatory effects of IAP antagonists likely involve activation of both the NIK-dependent NF-κB pathway and the activation of M1 type macrophages.

## Concluding Remarks

Tumor necrosis factor receptor-associated factor proteins, particularly TRAF6 and TRAF2, have been established as pivotal mediators of NF-κB and MAPK pathways. In addition, recent studies have identified TRAF2 and TRAF3 as negative regulators of the noncanonical NF-κB pathway and proinflammatory TLR signaling pathways. Accumulating studies have also revealed potential molecular mechanisms that regulate the fate and signaling functions of TRAFs. These research progresses provide novel insights into the mechanism underlying the signaling function of TRAFs and shed light on the molecular basis of human diseases associated with TRAF-dependent signaling pathways. These scientific advances have also suggested new opportunities for designing therapeutic approaches in the treatment of inflammatory diseases and cancer. For example, the IAP antagonists, which induce degradation of cIAPs and, thereby, disruption of the TRAF–cIAP E3 complex, have been tested in animal models of cancer immunotherapy and obtained promising results.

The recent progresses also raise a number of questions to be addressed in future studies. For example, the molecular mechanism by which TRAF6 mediates activation of NF-κB and MAPK pathways is incompletely understood. Although the E3 ligase activity of TRAF6 is important, controversies exist regarding how TRAF6 exerts the ubiquitin-dependent mechanism for activating the NF-κB and MAPK pathways. The signaling mechanism of TRAF2 is also elusive. In particular, whether the E3 ligase activity TRAF2 is required for TNFα-induced NF-κB activation is in debate. Furthermore, our current knowledge is largely based on cell line studies, and it will be important to have a mouse model for studying the role of ubiquitination in TRAF2 function under physiological conditions. Another important question is about the negative roles of TRAFs in regulating signaling. It is important to further define the mechanism by which TRAF2 and TRAF3 function together with cIAPs to mediate ubiquitin-dependent protein degradation and characterize the target proteins of this ubiquitin ligase complex. Finally, how TRAF proteins are regulated in different receptor pathways represents another important area for future research.

## Author Contributions

Both authors have made significant contributions to this review.

## Conflict of Interest Statement

The authors declare that the research was conducted in the absence of any commercial or financial relationships that could be construed as a potential conflict of interest. The reviewers HH and WZ and the handling Editor declared their shared affiliation.

## Abbreviations

TNFR, tumor necrosis factor receptor; TRAF, tumor necrosis factor receptor-associated factor; NF-κB, nuclear factor κB; IκB, inhibitory κB; IKK, IκB kinase; TAK1, transforming growth factor beta-activated kinase 1; NIK, NF-κB-inducing kinase; BAFFR, BAFF receptor; LTβR, lymphotoxin β receptor; ERK, extracellular signal-regulated kinase; JNK, c-Jun N-terminal kinase; MAPK, mitogen-activated protein kinase; MKK, MAPK kinase; MAP3K, MAPK kinase kinase; PRR, pattern-recognition receptor; TLR, toll-like receptor; NZF, Npl4 Zinc Finger; IL-1R, IL-1 receptor; IRAK, IL-1R-associated kinase; RANK, receptor activator of nuclear factor kappa-B; MAVS, mitochondrial antiviral signaling protein; MYSM1, myb-like SWIRM and MPN domain 1; NLR, NOD-like receptor; TRADD, TNF receptor-associated death domain; RIP1, receptor-interacting protein kinase 1; S1P, sphingosine-1-phosphate; SphK1, sphingosine kinase 1; DSS, dextran sodium sulfate; IBD, inflammatory bowel disease; CNS, central nervous system; EAE, experimental autoimmune encephalomyelitis; NDR1, Nuclear Dbf2-related kinase 1; IRF5, interferon regulatory factor 5.
